# Addition of Multi‐Level Technologies to Evaluate Eggs and Embryos and Improve Endometrial Quality Applied for In Vitro Fertilization to Achieve Pregnancy: A Case Study

**DOI:** 10.1002/ccr3.72149

**Published:** 2026-03-09

**Authors:** Tania G. Rojas‐Pérez, Yúvika Reyes‐Albarracín, Ginna Ortiz, Juan José Suárez, Diego Vilchis Reyes, Jacqueline Cordero Oropeza, Dinorah Hernández‐Melchor, Esther López‐Bayghen

**Affiliations:** ^1^ Instituto de Infertilidad y Genética SC INGENES México City Mexico; ^2^ Departamento de Toxicología Centro de Investigación y de Estudios Avanzados del Instituto Politécnico Nacional México City Mexico; ^3^ Instituto Regenera SC México City Mexico

**Keywords:** cumulus cells, endometrium regeneration, insulin resistance, PGTA, platelet‐rich plasma, transcription

## Abstract

Preimplantation genetic testing for aneuploidies (PGTA) diagnoses chromosomal abnormalities and can be complemented by cumulus cell transcriptional analysis to assess egg and ovarian quality and development during embryo culture. In addition to selecting the best available embryo, intrauterine administration of platelet‐rich plasma (PRP) and correction of insulin resistance (IR) through nutritional intervention can improve endometrial quality. This case report examines the potential feasibility of combining in vitro fertilization (IVF) with four techniques to achieve a viable pregnancy. A couple with a prior successful IVF pregnancy was seeking a second pregnancy. The patient was diagnosed with polycystic ovary syndrome (BMI = 24.5 kg/m^2^), whereas her partner had teratozoospermia. For the first IVF cycle, using PGTA, we confirmed that only euploid embryos were transferred. A hysteroscopic examination confirmed a lack of endometrial competence, and treatment with autologous intrauterine PRP improved endometrial receptivity; however, after a single‐embryo transfer, the embryo did not implant. The patient underwent a nutritional intervention to improve insulin resistance with a low‐carb dietary intervention (carbohydrates < 50 g/day, protein 1.5 g/day) that reduced insulin resistance after 2 weeks. For the next IVF cycle, the low‐carb dietary intervention was continued, along with another PRP application, and the cumula cells transcriptional analysis was performed in addition to PGTA, identifying two optimal euploid embryos. This time, embryo implantation occurred (β‐hCG: 362.0 mIU/mL). The pregnancy ended at 39 weeks, with a healthy male baby (length: 53 cm; weight: 3465 g; Apgar 8/9). In conclusion, using molecular techniques for embryo selection (cumula analysis and PGTA) and interventions to improve endometrial function (PRP and a dietary intervention) are an effective strategy that strengthens IVF and increases the likelihood of achieving a viable pregnancy.

## Key Clinical Message

Personalized regenerative strategies coupled with a systematic molecular diagnosis are a powerful combination to improve endometrial quality and embryo selection for achieving pregnancy in multi‐causal infertile patients using their ova.

## Graphical abstract

This integrated clinical approach optimizes the reproductive cycle in several steps, starting with nutrition (A): restricting carbohydrates and inducing ketosis to reduce inflammation and improve insulin sensitivity, which benefits follicular development. Clinicians then assess metabolic priming success by measuring the PVL index in granulosa cell via qRT‐PCR (B), providing insight into oocyte health. After fertilization, embryos undergo PGT‐A analysis (C) to check for chromosomal abnormalities, selecting only normal (euploid) embryos for transfer. Finally, Platelet‐Rich Plasma (D) is applied to the endometrium, delivering growth factors that enhance uterine tissue and vascularity, creating an optimal environment for implantation.

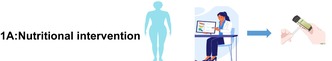

## 1 Introduction

Reproductive medicine faces the challenge of diagnosing and resolving problems at multiple levels within a single case. From oocyte collection, embryo fertilization, and selection to improving patient health for implant preparation and development, this work still needs to be done individually or, ideally, collectively. Preimplantation genetic testing for aneuploidies (PGTA) screens for chromosomal abnormalities and enables the selection of euploid embryos, thereby increasing the likelihood of obtaining normal embryos, potentially limiting diseases such as Turner and Laron Syndromes, propionic acidemia, and Carnitine‐acylcarnitine Translocase Deficiency [1, 2, 3, 4]. In addition to genomic testing, noninvasive alternatives are still needed to assess embryonic parameters and enhance the potential for successful implantation. For example, analyzing cumulus cells (CC) as a non‐invasive way to assess individual egg quality through transcriptional analysis of Prostaglandin‐Endoperoxide Synthase 2 (*PTGS2*) and Versican (*VCAN*) gene expression, normalized to the L19 ribosomal gene as a control (PVL index). A PVL index score ≥ 58.2 has been associated with oocytes that produce better embryos for transfer [5]. These two techniques could more accurately select metabolically competent oocytes and euploid embryos.

Concerning the maternal side, an excellent endometrial lining is not always achievable. It is challenging to diagnose as fit—ideally, 7 mm thick at embryo implantation with a trilaminar structure [6]. Endometrial regeneration with endogenous growth factors involves applying platelet‐rich plasma (PRP) during endometrial preparation to improve endometrial quality in infertile patients [7]. Alternatively, improving the patient's health through nutritional interventions that correct their metabolic profile and insulin resistance (IR) could improve in vitro fertilization (IVF). In patients with polycystic ovarian syndrome (PCOS), a low‐carb dietary intervention (limited carbohydrate intake) improved waist circumference, serum triglycerides, and fasting plasma glucose (FPG), leading to reduced IR and favoring implantation and clinical pregnancy [8]. Here, we present the case of a patient who underwent four combined techniques and achieved 39 weeks of pregnancy, resulting in the delivery of a male baby.

## 2 Case Presentation

### 2.1 Case History, Physical, and Laboratory Examinations

A couple with secondary infertility associated with PCOS and teratozoospermia attended the Instituto de Infertilidad y Genética México SC, INGENES, México City, in 2023. The couple opted to undergo IVF due to a successful IVF in 2019, where three euploid embryos were transferred. This earlier IVF (2020) resulted in a healthy male baby.

For the 35‐year‐old male partner, sperm quality was obtained from the Andrology Laboratory of the Ingenes Institute. Semen samples were collected by masturbation (after 3–5 days of abstinence) on the day of the IVF procedure. After liquefaction, samples were analyzed according to the WHO criteria for volume, time of liquefaction, pH, leukocyte concentration, sperm concentration, progressive motility, morphology (by the Kruger criteria), and viability according to the WHO (2010) parameters [9, 10]. Sperm morphological diagnosis indicated abnormal shapes (teratozoospermia).

The 36‐year‐old female was slightly overweight (BMI = 25.4 kg/m^2^). Her overall medical history was unremarkable, with no reproductive health conditions. The patient's anthropometric measurements were within standards for Latina women. The patient had a history of PCOS. Imaging examination showed more than 30 follicles on each ovary, as detected by ultrasound. PCOS was confirmed using the Rotterdam criteria: at least two of three conditions: (a) clinical signs of hyperandrogenism, (b) oligo/amenorrhea or amenorrhea, and (c) polycystic ovaries confirmed by ultrasound [11]. Laboratory tests were performed to evaluate metabolic status. In the initial evaluation, the patient was in a state of hyperinsulinemia. HOMA‐IR index was calculated, indicating she was IR.

**FIGURE 1:**
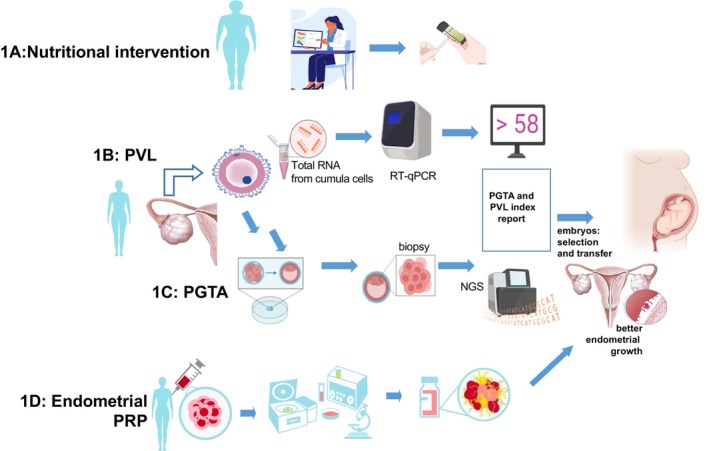
Representative and schematic overview of all technologies applied together in the case. (A) Nutritional intervention based on carbohydrate restriction and ketosis induction. (B) Granulosa cells qRT‐PCR analysis to analyze PVL index. (C) PGT‐A analysis to determine genomic normality in chromosomal number. (D) Plasma‐rich platelet applied to the endometrium before endometrial preparation to improve endometrial growth.

For this case study, four methods were employed: (1) nutritional intervention to improve critical metabolic factors, such as FPG, triglycerides (TG), and insulin (FPI); (2) intrauterine PRP application to improve endometrium quality and promote embryo implantation; (3) assessing oocyte quality with the PVL index; and (4) PGTA to ensure only euploid embryos were transferred (Figure 1).

#### 2.1.1 IVF

The patient underwent controlled ovarian stimulation with a gonadotropin‐releasing hormone antagonist (Gonal 150 UI and Merapur 150 UI). The stimulus was prolonged until the diameter of the leading follicles was > 18 mm. Afterward, recombinant human chorionic gonadotropin was administered. After 36 h, the oocytes were retrieved with ultrasound guidance. All 14–18 mm follicles were aspirated, and ova were collected and fertilized by intracytoplasmic sperm injection (ICSI) with the partner's sperm and then cultured. Embryos were cryopreserved using vitrification.

Frozen–thawed embryos were implanted during an estrogen‐primed cycle. The uterine transfer occurred during a controlled endometrial development cycle free of gonadotropins. Endometrial preparation with 6 mg of estrogen valerate (Primogyn, UPC: 00770333115702) was started on menstrual cycle day (MCD) 2 with a 10‐day increasing‐dose scheme, beginning with 2 mg/day and increasing to a maximum of 8 mg/day, and assessing endometrial mean thickness (EMT) every other day via ultrasound. Blastocysts were transferred. Embryo implantation was confirmed on Day 14 by serum β‐hCG concentrations > 10 mUI/mL and clinical pregnancy by the presence of a fetal heartbeat using ultrasound at 6 weeks.

#### 2.1.2 Preimplantation Genetic Test for Aneuploidies (PGTA)

Aneuploidy analysis was performed according to the Ingenes Institute's standardized protocol, using trophectoderm biopsies from morphologically good‐quality embryos before vitrification [12].

#### 2.1.3 Intrauterine PRP

Autologous PRP administration was performed according to Hernandez‐Melchor et al. [7] into the uterus during an estrogen‐primed cycle. Briefly, venous blood (3 mL) was drawn from the patient and centrifuged at 1200 rpm for 12 min. The plasma was obtained and centrifuged again at 3300 rpm for 7 min. PRP was collected and infused into the uterus within 10 min. On MCD 10, EMT was measured using ultrasonography, and 1 mL of autologous PRP was collected and infused into the uterine cavity within 10 min of isolation to avoid protein degradation, using a Wallace Embryo Transfer Catheter (SureView Soft 2 CE123). The procedure was repeated on MCD 12 as the EMT was < 7 mm.

#### 2.1.4 Dietary Assessment and Nutritional Intervention

Using a direct food intake questionnaire, the patient's carbohydrate, fat, and protein intake was calculated in the MyFitnessPal app (before and during the nutritional intervention). The nutritionist confirmed the daily and weekly macronutrient intake and corrected the diet's macronutrient distribution when needed. A low‐carb dietary intervention, averaging approximately 1800 cal with a maximum daily carbohydrate intake of 50 g, was advised, with macronutrients adjusted to 20% carbohydrates, 35% protein, and 40% fat. Ketosis was monitored using Medicon Ketone Test Strips (China), and urinary ketones > 40 mg/dL were considered positive for ketosis. The patient received individualized counseling focused on achieving adequate intake of essential nutrients while limiting carbohydrates to levels that support stable glycemia and control of hyperinsulinemia. The dietary structure emphasized three main meals per day. Meal composition included a high‐quality protein source (such as fish, poultry, eggs, lean meats, or unsweetened dairy), non‐starchy vegetables, and healthy fats (such as olive oil, avocado, nuts, and seeds), as needed to support satiety and reduce postprandial glycemic excursions. At the same time, intake of ultra‐processed foods, refined carbohydrates, added sugars, grains, and foods high in sugar was reduced to a minimum.

Analysis for FPG, TG, and FPI was performed at an outside facility. Insulin sensitivity was calculated according to the QUICKI index: 1/(log FPG + log FPI), in which a value < 0.357 is considered low insulin sensitivity. IR was calculated according to the HOMA‐IR index: (FPG*FPI)/405, in which a score of > 2.0 is IR. The triglyceride‐glucose‐BMI (TyG‐BMI) index was calculated as ln(TG × FPG/2) × BMI.

#### 2.1.5 PVL Index

The PVL index was determined using the method described by Ocampo et al. [5]. Briefly, the CCs were mechanically separated and collected for each cumulus‐oocyte complex. Afterward, the CCs' total RNA was isolated using TRIzol reagent according to the manufacturer's protocol. The expression, as a function of the cycle threshold, for the *PTGS2* and *VCAN* genes, normalized to the *L19* ribosomal gene, was measured by quantitative reverse transcription‐polymerase chain reaction to obtain the PVL index: [(*PTGS2* + *VCAN*)*L19] [5]. The Step One Plus apparatus (Applied Biosystems) with the One Step Kappa Syberfast system (KAPA Biosystems, Woburn, MA, USA) was used in this study. A score ≥ 58.2 is a probable embryo of adequate quality for transfer.

## 3 Results

**FIGURE 2:**
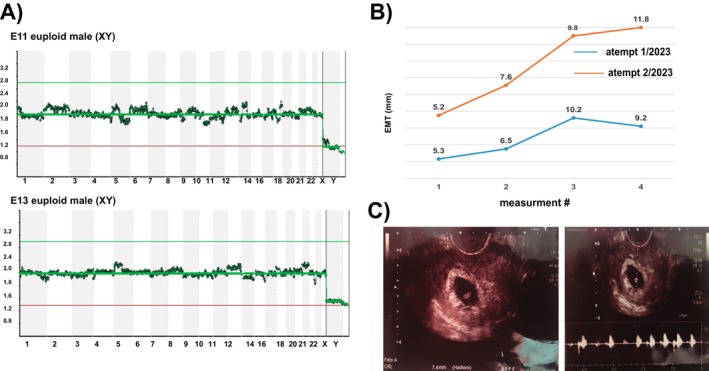
(A) PGT‐A screening via genomic analysis for the selection of euploid embryos. (B) The endometrial mean thickness, along with the two major interventions. Endometrial thickness after regeneration using PRP to improve endometrial quality, achieving good endometrial growth in the two attempts; measurements after PRP application and endometrial preparation for frozen–thawed embryo transfer cycles are shown. On the first attempt in 2023, blue, one embryo diagnosed as euploid, was negative for pregnancy. In the second embryo transfer, after correcting the insulin resistance characteristic of the PCOS patient, endometrial thickness is shown by the orange line: 11.8 mm at the time of transfer. (C) Confirmation of clinical pregnancy by ultrasound showing a single embryonic sac with a fetal heartbeat. The sonogram is 6 weeks after embryo transfer.

Initial evaluation determined that the patient suffered from PCOS with IR. Chronologically, the patient and her partner attended Ingenes for their second IVF. They underwent a standard protocol. Lack of endometrial competence was suspected and confirmed by hysteroscopy, but no abnormalities were found. The EMT was suboptimal, and a thin endometrium was diagnosed (before hysteroscopy, EMT = 3.6 mm). PRP was infused to improve endometrial quality before the embryo transfer (ET) attempt. Endometrial preparation was delayed by 1 cycle to achieve adequate endometrial growth (Figure 2B). The PRP treatment improved EMT thickness, and one euploid embryo was transferred (PGTA‐confirmed) but did not implant (β‐hCG < 10 mUI/mL). The patient opted to postpone another IVF cycle to lose weight.

By the patient's efforts, she lost weight and reduced her BMI to 20.3 kg/m^2^. Nevertheless, IR was assessed (HOMA‐IR), and even with the decrease in BMI, metabolic abnormalities persisted. The patient typically consumed an unbalanced standard American 2000‐cal diet with 55% carbohydrates, 25% fat, and 20% protein content. The patient was advised to follow a ketogenic diet to correct IR. Within 2 weeks, a 2‐kg weight loss and a reduction in BMI were observed (Table 1). Decreases were also observed in FPG and TG, lowering HOMA‐IR below the 2.0 threshold while increasing insulin sensitivity (Table 1).

**TABLE 1:** Descriptive of the case and resulting parameters after the combination of complementary techniques.

Category	Failed IVF treatment 2023	Successful IVF treatment to achieve a second pregnancy in 2023	
Nutritional intervention	No	Ketogenic diet	
Anthropometric/nutritional parameters			
		Nutritional intervention	
		Before	After
Age (years)	36	36	
Weight (kg)	53	52	50
Waist circumference (cm)	NA	72	69
BMI (kg/m^2^)	25.4	20.3	19.5
Blood pressure (mmHg)	N/A	100/60	90/60
Carbohydrates (g/day)	NA	176	49
Biochemical parameters			
Fasting glucose (mg/dL)	NA	77	75
Ketones in urine (mg/dL)	NA	Negative	80
Insulin (mIU/mL)	NA	15.9	8.57
HOMA‐IR	NA	3.02	1.59
QUICKI (insulin sensitivity)	NA	0.324	0.356
Triglycerides (mg/dL)	NA	123	94
Triglycerides‐glucose‐BMI	NA	171.8	159.27
IVF parameters			
Oocyte source	Patient	Patient	
Oocytes collected (*n*)	26	27	
Oocytes fertilized (%)	76%	59%	
Embryos (*n*)	4	5	
Molecular analysis			
PGTA	**E1 Normal XY** E3 Abnormal (−22) XX E4 Abnormal (−8) XX E11 Abnormal (−16, +22) XX	**E11 Normal XY** **E13 Normal XY** **E15 Abnormal (−1, −4) XX** **E21 Abnormal (+16) XY** **E27 Normal XY**	
PVL (transcriptional analysis of cumulus cells)	NA	**E11 = PVL 68.89** **E13 = PVL 67.31** E15 = PVL 51.19 E21 = PVL 52.28 **E27 = PVL 59.64**	
Embryo quality (morphology grade at inner cells mass/trophectoderm)	**E1 B/B** E3 B/B, E4 B/B, E11 A/B	**E11 B/B** **E13 B/C**	
Embryos transferred	1 (E1)	2 (E11, E13)	
Endometrial interventions			
Endometrial corrections^a^	Hysteroscopy (normal cavity) and platelet‐rich plasma (two applications)		
Endometrial thickness (mm; at the transfer day)	9.2	11.8	
Obstetric outcomes			
Implantation	Negative	Positive	
β‐hCG day 14	NA	362.0 mUI/mL	
Clinical pregnancy (US result)	NA	Yes (one sack with heartbeat)	
Nutritional control during pregnancy	NA	54.2 g average CH/day	
Pregnancy result	NA	39 weeks, healthy male, 53 cm, 3465 g, Apgar 8/9	

*Note:* Bold values indicate embryos showing best quatlity using PVL and PGT‐A.

Abbreviation: NA, not applicable.

^a^ Data have shown that the endometrial improvement achieved with this technique increases progressively in the following 3 months after PRP application (6).

The patient underwent a second round of controlled ovarian stimulation. However, this time, using the CCs, the PVL index was generated. Three oocytes were identified as potentially high‐quality (11, 13, and 27). After ISCI fertilization, embryos 11 and 13 were confirmed as euploid. PRP was infused to improve endometrial quality before ET. USG of the endometrium confirmed the ideal trilaminar structure (EMT = 11.8 mm), and the two embryos were transferred. Table 1 depicts the yields and quality of the embryos, whereas Figure 2A shows the euploidy of the two male‐transferred embryos. For the second ET, IR was corrected (Table 1). Ultrasound confirmed a clinical pregnancy with an embryo sac, fetal heartbeat of 131 beats per minute at Week 6 with normal amnio, and absence of markers for chromosomal defects (Figure 2C). The pregnancy was monitored through a structured prenatal care program under maternal–fetal medicine, with follow‐up visits scheduled once a month. The care protocol includes a first‐trimester screening between 11 and 14 weeks of gestation, during which maternal serum markers and an early ultrasound assessment were performed to evaluate the risk of chromosomal abnormalities and to assess early fetal development, yielding results within the normal range. A comprehensive second‐trimester anatomical (structural) scan was performed at 23 weeks to evaluate fetal anatomy in detail, screen for congenital anomalies, assess placental and uterine conditions, and confirm the male fetus. These follow‐ups monitor maternal well‐being, fetal growth, amniotic fluid volume, and other parameters essential to ensuring a healthy pregnancy. This schedule was continued until delivery, resulting in a healthy boy at Week 39 (length: 53 cm; weight: 3465 g; Apgar 8/9).

## 4 Discussions

In this case, multi‐level technologies were combined at the maternal and embryo levels, evaluating oocytes and embryos and improving endometrial quality to achieve a successful pregnancy.

Patients with PCOS exhibit lower pregnancy rates due to the inherent reproductive complications associated with the IR. A low‐carb dietary intervention was implemented to address IR associated with the PCOS condition. Clinical evidence has shown that patients with PCOS and IR exhibit poor oocyte development and maturation, particularly affecting the early stages of IVF cycles [13, 14]. Also, there is a higher proportion of adverse obstetric outcomes despite good oocyte quality and euploid embryos, suggesting that one adverse effect of IR may directly affect endometrial tissue and embryo implantation [15]. IR begins to develop due to excessive carbohydrate consumption, leading to a state of chronic hyperinsulinemia and a progressive loss of insulin sensitivity. In this context, restricting excessive carbohydrate consumption (glucotoxicity) was associated with correction of IR and improved obstetric outcomes [8, 14]. After low‐carb dietary intervention, the final HOMA‐IR, QUICKI, and TyG‐BMI indices indicated that IR resolved, whereas insulin sensitivity increased. The mechanism by which IR affects the endometrium is increasingly well described, and it is associated with the tissue's high energy demand. Any alteration in glucose and insulin homeostasis will disrupt endometrial cell growth and development, decidualization, and blastocyst recognition and adhesion, potentially leading to embryo implantation failure due to IR. Liver concentrations of Insulin‐like Growth Factor Binding Protein 1 and Sex Hormone Binding Globulin lead to decreased GLUT4 transporter‐dependent glucose uptake, contributing to hyperandrogenism [15] and exacerbating inflammation [6, 16, 17]. Endometrial tissue regeneration in patients with PCOS appears necessary to achieve positive obstetric outcomes. Although this patient had previously improved (EMT > 7 mm) with the PRP therapy, pregnancy has not been achieved even when transferring an euploid embryo. Endometrial fitness improved following the intensive nutritional intervention at the time of ET, with a final EMT of 11.8 mm. Regarding the maternal side, endometrial fitness improved following PRP and intensive nutritional intervention. Both may have equally contributed to embryo implantation and pregnancy development.

The PVL index was used to assess oocyte quality by analyzing CC transcriptional profiles. This index can predict the embryo's implantation potential by identifying a higher capacity to implant, leading to a clinical pregnancy [5, 18]. A high PVL index is associated with higher clinical pregnancy rates, as the assessed genes play a crucial role in oocyte maturation and are relevant indicators of oocyte competence [5]. In the previous cycle for this patient, four embryos progressed to day 5 of development (Table 1); however, PGTA analysis revealed that only one was euploid. Five mature embryos were obtained in the subsequent cycle, of which three were euploid and showed the best PVL indices (E11 = 68.89, E13 = 67.31, E27 = 59.64). The patient and clinician decided to transfer the male embryos E11 and E13. The use of the PVL index, coupled with standard morphological evaluation and PGTA, contributed to the success of the second pregnancy. The PVL index provides an alternative method for discriminating embryos from the same patient based on genetic parameters.

PCOS is known for being more difficult to keep a pregnancy, as it is associated with an increased risk of miscarriage, gestational diabetes mellitus, hypertensive disorders of pregnancy, preterm delivery, and gestational growth retardation [19, 20, 21]. Ranging from 200 to 350 g of daily carbohydrates, around 55% of the overall caloric intake from the typical American diet, the significant harms of refined carbohydrates occur from consuming excessive amounts of sugar‐laden foods. Elevated intakes of sugar‐laden foods are associated with an increased prevalence of metabolic syndrome and obesity (44%) and a higher risk of developing diabetes (26%) [22]. Evidence indicates that nutritional status influences endometrial receptivity and implantation. Diets characterized by controlled carbohydrate intake, low glycemic load, and nutrient‐dense food choices stabilize insulin levels and reduce inflammation and may promote a better peri‐implantation environment [23, 24]. In addition, improving metabolic health prior to an in vitro fertilization cycle could optimize the endometrial environment and potentially increase the likelihood of implantation, although additional mechanistic and interventional studies are needed [23, 24, 25].

An additional follow‐up of the patient's nutritional habits, using a monthly questionnaire, revealed that the patient maintained a low‐carbohydrate intake, averaging 75 g/day, and primarily avoided sugary and overprocessed foods, while consuming more vegetables as the primary carbohydrate source (Table 2).

**TABLE 2:** Resulting parameters following carbohydrate care during pregnancy.

	^a^	^b^	Pregnancy week				
			6	8	13	17	28
Carbohydrates (g/day)	49	93	59	48	55	57	52
Weight (kg)	50.2	50	49	48.7	48	55.5	57
BMI (kg/m^2^)	21.1	23.1	22.7	22.5	22.2	25.7	26.4
Fasting glucose (mg/dL)	75	81	84	81	74	81	76
Ketones in urine (mg/dL)	80	NA	NA	NA	NA	NA	NA
Insulin (mIU/mL)	8.57	NA	9.1	NA	NA	11	NA
HOMA‐IR	1.59	NA	1.88	NA	NA	2.2	NA
QUICKI	0.356	NA	0.347	NA	NA	0.339	NA
Triglycerides (mg/dL)	94	96	100	84	61	96	109
Triglycerides‐glucose‐BMI	159.2	161.1	159.7	155.4	147.2	153.4	185.4

^a^ Data at the end of the nutritional intervention and before starting a new controlled ovarian stimulation.

^b^ Data taken 2 days after second transfer in 2023.

There is a need for further research evaluating the effects of a low‐carb dietary intervention during pregnancy on child development. The low‐carb dietary intervention may have a positive effect on fertility in women with PCOS and blood pressure levels during pregnancy or take part in the prevention and treatment of diseases such as epilepsy, obesity, asthma, depression, anxiety, or autism.

## 5 Conclusions

The effectiveness of IVF in achieving a viable pregnancy is strengthened by jointly using techniques for embryo selection, IR correction, and endometrial regeneration. The low‐carb dietary intervention and endometrial regeneration increased endometrial thickness, resulting in a healthy birth.

## Author Contributions

**Tania G. Rojas‐Pérez:** conceptualization, data curation, formal analysis, investigation, methodology. **Yúvika Reyes‐Albarracín:** data curation, investigation, methodology. **Ginna Ortiz:** investigation, methodology. **Juan José Suárez:** investigation, methodology. **Jacqueline Cordero Oropeza:** investigation, methodology. **Diego Vilchis Reyes:** investigation, methodology. **Dinorah Hernández‐Melchor:** data curation, writing – review and editing. **Esther López‐Bayghen:** conceptualization, investigation, project administration, resources, supervision, writing – original draft, writing – review and editing.

## Funding

The authors have nothing to report.

## Ethics Statement

The patient signed an informed consent form, and all identifiable data were anonymized in accordance with the Declaration of Helsinki and local clinical research regulations.

## Consent

The patient provided written informed consent to publish this report in accordance with the journal's patient consent policy. The patient and her partner also provided written informed consent to participate in this study, in accordance with the Declaration of Helsinki and to the publication of their anonymized information in this article.

## Conflicts of Interest

The authors declare no conflicts of interest.

## Data Availability

Data are available under request.
